# TGF-β1-induced EMT promotes targeted migration of breast cancer cells through the lymphatic system by the activation of CCR7/CCL21-mediated chemotaxis

**DOI:** 10.1038/onc.2015.133

**Published:** 2015-05-11

**Authors:** M-F Pang, A-M Georgoudaki, L Lambut, J Johansson, V Tabor, K Hagikura, Y Jin, M Jansson, J S Alexander, C M Nelson, L Jakobsson, C Betsholtz, M Sund, M C I Karlsson, J Fuxe

**Affiliations:** 1 Division of Vascular Biology, Department of Medical Biochemistry and Biophysics, Karolinska Institute, Stockholm, Sweden; 2 Department of Chemical and Biological Engineering, Princeton University, Princeton, NJ, USA; 3 Department of Microbiology, Tumor and Cell Biology, Karolinska Institutet, Stockholm, Sweden; 4 Division of Cell Regeneration and Transplantation, Department of Functional Morphology, Nihon University School of Medicine, Tokyo, Japan; 5 Department of Surgical and Perioperative Sciences/Surgery, Umea University, Umea, Sweden; 6 Department of Molecular and Cellular Physiology, Louisiana State University Health Sciences Center, Shreveport, LA, USA

## Abstract

Tumor cells frequently disseminate through the lymphatic system during metastatic spread of breast cancer and many other types of cancer. Yet it is not clear how tumor cells make their way into the lymphatic system and how they choose between lymphatic and blood vessels for migration. Here we report that mammary tumor cells undergoing epithelial–mesenchymal transition (EMT) in response to transforming growth factor-β (TGF-β1) become activated for targeted migration through the lymphatic system, similar to dendritic cells (DCs) during inflammation. EMT cells preferentially migrated toward lymphatic vessels compared with blood vessels, both *in vivo* and in 3D cultures. A mechanism of this targeted migration was traced to the capacity of TGF-β1 to promote CCR7/CCL21-mediated crosstalk between tumor cells and lymphatic endothelial cells. On one hand, TGF-β1 promoted CCR7 expression in EMT cells through p38 MAP kinase-mediated activation of the JunB transcription factor. Blockade of CCR7, or treatment with a p38 MAP kinase inhibitor, reduced lymphatic dissemination of EMT cells in syngeneic mice. On the other hand, TGF-β1 promoted CCL21 expression in lymphatic endothelial cells. CCL21 acted in a paracrine fashion to mediate chemotactic migration of EMT cells toward lymphatic endothelial cells. The results identify TGF-β1-induced EMT as a mechanism, which activates tumor cells for targeted, DC-like migration through the lymphatic system. Furthermore, it suggests that p38 MAP kinase inhibition may be a useful strategy to inhibit EMT and lymphogenic spread of tumor cells.

## Introduction

Lymph metastasis is the earliest sign of metastatic spread and the most powerful prognostic factor in breast cancer.^1, 2^ Lymph vessel invasion may be a better prognostic marker in breast cancer compared with blood vessel invasion.^3^ Unlike blood vessels, lymphatic vessels are equipped with unique button-like junctions that support entry of both fluid and dendritic cells (DCs) into the lymphatic system.^4^ Thus, there may be a structural-based prerequisite for migratory tumor cells to intravasate into lymphatic vessels rather than blood vessels within the tumor microenvironment. However, it is not clear how tumor cells find their way to lymphatic vessels and whether this is a regulated or more of a stochastic process.

Breast cancer progression toward invasive and metastatic disease is associated with the reactivation of epithelial–mesenchymal transition (EMT), a latent developmental process, which involves transdifferentiation of epithelial cells into mesenchymal-like cells with migratory and stem cell properties.^5, 6, 7, 8, 9^ Transforming growth factor-β (TGF-β) is a potent inducer of EMT both during development and in cancer.^10, 11, 12^ Elevated levels of TGF-β1 have been found in plasma of breast cancer patients and at invasive fronts in human breast cancer tissues, and correlate with the presence of lymph node metastasis.^13, 14^ Immune cells, such as macrophages and regulatory T cells, represent cellular sources of TGF-β1 in the tumor microenvironment.^15^ Thus, TGF-β-induced EMT represents a link between cancer and inflammation. Along these lines, recent data indicate that breast cancer cells undergoing EMT acquire immune cell properties.^15, 16^

TGF-β signaling toward EMT is mediated by both Smad-dependent and Smad-independent pathways, including p38 MAP kinase (p38 MAPK). Although the Smad pathway is unique to TGF-β signaling, p38 MAPK can also be activated by other pathways including Ras and Wnt, which cooperate with TGF-β to induce EMT.^10, 12, 17^ The EMT response downstream of TGF-β signaling is induced by transcriptional reprogramming, which promotes inactivation of genes encoding epithelial proteins, such as E-cadherin and other junction proteins, and activation of genes encoding mesenchymal proteins including N-cadherin and vimentin.^10, 11, 12, 18^ As a result, tumor cells undergoing TGF-β-induced EMT acquire the capacity to detach and migrate away from the primary tumor.

Recently, TGF-β signaling was shown to promote single-cell migration of mammary tumor cells.^19^ However, it is not clear whether EMT cells make use of their enhanced migratory capacity to migrate in a random or, alternatively, in a more targeted fashion. We used a syngeneic mouse model in combination with a three-dimensional (3D) co-culture model to test the hypothesis that TGF-β1-induced EMT promotes targeted migration of tumor cells toward lymphatic vessels.

## Results

### TGF-β-induced EMT promotes lymphatic dissemination of mammary tumor cells

**Figure 1:**
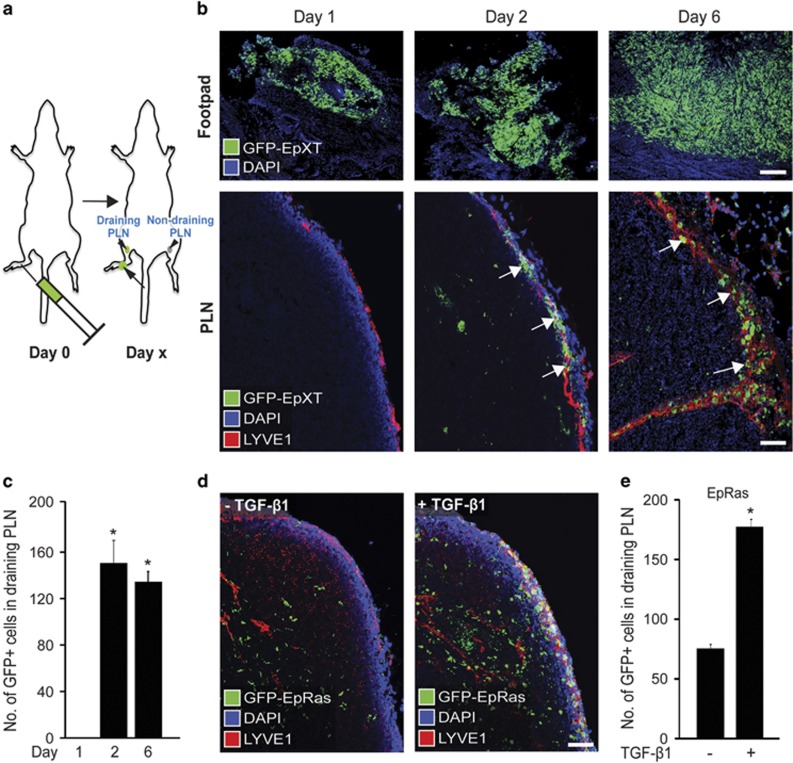
TGF-β1-induced EMT promotes lymphatic dissemination of mammary tumor cells. (**a**) Schematic drawing of the footpad model used to study the effect of TGF-β1-induced EMT on lymphatic dissemination of mouse mammary tumor cells in syngeneic BALB/c mice. The sites of injection (syringe), primary tumor growth (arrow), draining (ipsilateral) and non-draining (contralateral) PLN (arrowheads) are indicated. (**b**) Representative confocal immunofluorescence images showing GFP-labeled EpXT (GFP-EpXT) cells at the site of injection in the footpad (upper panel), and in draining PLN (lower panel) at 1, 2 and 6 days after injection. Arrows mark GFP-EpXT cells detected in subcapsular sinuses of PLN at day 2 and day 6 after injection. Scale bars, 200 μm (footpad images) and 50 μm (PLN images). (**c**) Bar graph showing quantification of GFP-EpXT cells that had migrated to PLN at day 1, 2 and 6 after injection in the footpad. (**d**) Confocal immunofluorescence images showing GFP-EpRas cells in draining PLN at 2 days after injection in the footpad. Before injection, EpRas cells had been either untreated (−TGF-β1, left panel), or pretreated for 14 days with 10 ng/ml of TGF-β1 (+TGF-β1, right panel). Scale bar, 50 μm. (**e**) Bar graph showing the quantification of GFP-EpRas cells in draining PLN at 2 days after injection into the mouse footpad. GFP-EpRas cells had been either untreated (−TGF-β1) or pretreated for 14 days with 10 ng/ml of TGF-β1. Calnexin was used as a loading control in immunoblotting experiments. *P<0.05.

To study whether the induction of EMT would affect tumor cell dissemination through the lymphatic system, we set up a mouse model frequently used to study trafficking of DCs to draining lymph nodes after the subcutaneous injection of cells into the hind footpad of syngeneic, recipient BALB/c mice (Figure 1a). Previous studies have shown that DCs migrate to draining popliteal lymph nodes (PLN) within 1–2 days after injection in the footpad.^20^ This model has also been used to study lymphatic dissemination of tumor cells, such as B16 melanoma cells.^21^

First, we used EpXT cells, which are stably in EMT through cooperative effects of TGF-β1 and oncogenic Ras signaling.^22^ In comparison with parental EpH4 cells, EpXT cells display EMT properties including an elongated cell shape and a low E-to-N-cadherin ratio (Supplementary Figures 1a and b). Green fluorescence protein (GFP)-labeled EpXT (GFP-EpXT) cells were injected into the footpads of BALB/c mice to study the capacity of these cells to migrate to draining PLN. Tumors of progressively increasing sizes were found at injection sites in the footpads at 1, 2 and 6 days after injection (Figure 1b). From day 2 and onwards, GFP-EpXT cells were also detected in draining PLN (Figure 1b, lower panel), but not in contralateral, non-draining PLN (data not shown). Both individual and small groups of GFP-EpXT cells were found in draining PLN and they were primarily located in the subcapsular sinuses indicating that they had entered through afferent lymphatic vessels. Quantification confirmed the presence of GFP-EpXT cells in draining PLN at days 2 and 6 after injection (Figure 1c). Injection of GFP-EpH4 cells also resulted in the formation of small tumors in the mouse footpad, but no GFP-EpH4 cells could be detected in lymph nodes up to six days after injection (Supplementary Figure 1c).

Next we studied whether the transient induction of EMT would affect the capacity of mammary tumor cells to migrate through the lymphatic system. For this, we used EpRas cells, which similar to EpH4 cells retain an epithelial morphology in culture (Supplementary Figure 1d). However, in comparison with EpH4 cells, EpRas cells expressed decreased levels of E-cadherin and increased levels of N-cadherin, indicating that Ras transformation initiated a partial EMT in these cells (Supplementary Figure 1e). Exposure to TGF-β1 (10 ng/ml) for 48 h resulted in more profound EMT as evidenced by cellular elongation, repression of E-cadherin and induction of vimentin and Snail (Supplementary Figures 1d and e). Long-term treatment with TGF-β1 for 14 days accentuated EMT even further and this EMT state was maintained for at least 6 days after TGF-β1 withdrawal (Supplementary Figure 1f). We performed footpad injections with GFP-EpRas cells and found that cells that had been pretreated with TGF-β1 for 14 days migrated significantly more efficient to PLN compared with nontreated cells (Figure 1e).

### EMT cells migrate toward lymphatic vessels

**Figure 2:**
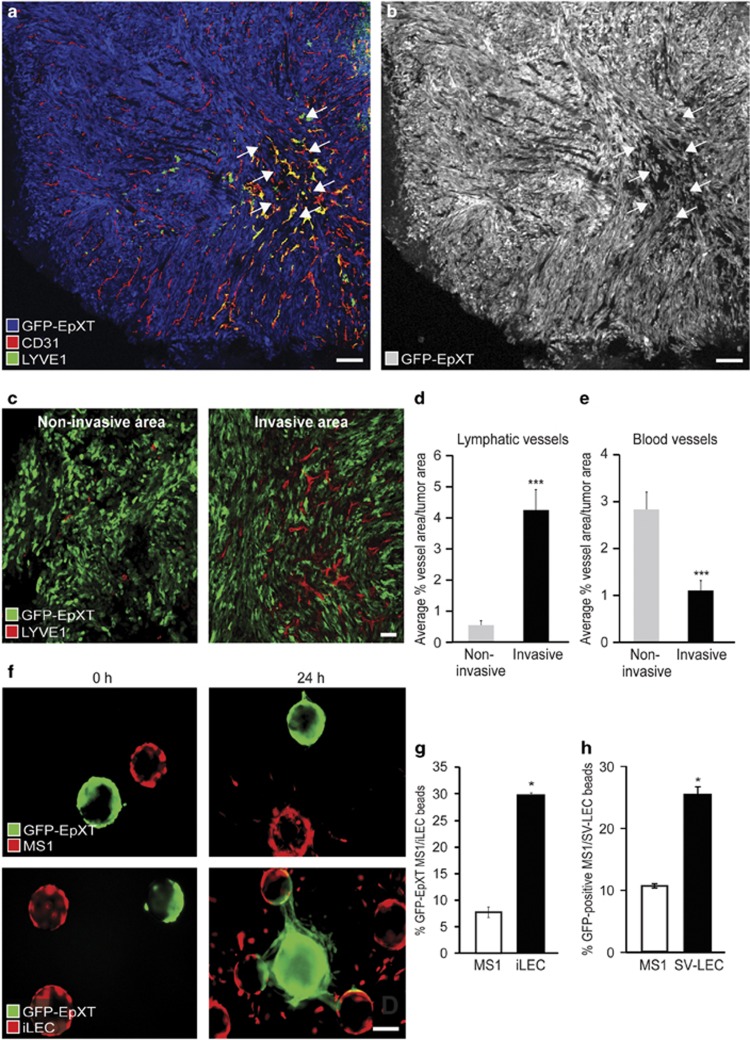
EMT cells migrate toward lymphatic vessels in experimental tumors and in 3D beads assays. (**a**) Representative confocal immunofluorescence images of GFP-EpXT footpad tumors at day 6 after injection. The image of the GFP-EpXT cells were moved from green channel and placed in the blue channel to allow for visualization of lymphatic vessels (green), and blood vessels (red), which are double-stained for LYVE-1 (only labels lymphatic vessels) and CD31 (labels both blood and lymphatic vessels). (**b**) Black and white image of GFP-EpXT cells. Invasive areas (I) containing elongated GFP-EpXT cells lining up in unidirectional patterns are indicated by arrows in both images. (**c**) Representative high magnification confocal images showing the presence of lymphatic vessels in noninvasive (left panel) versus invasive areas (right panel) GFP-EpXT tumors. (**d** and **e**) Bar graphs showing results from quantification of area density of lymphatic vessels (**d**) and blood vessels (**e**) in noninvasive versus invasive areas to GFP-EpXT footpad tumors. (**f**) Immunofluorescence images showing polymeric beads coated with GFP-EpXT cells (green) and co-cultured in a 3D fibrin matrix with beads coated with DsRed-labeled MS1 vascular endothelial cells (DsRed-MS1; upper panels), or immortalized lymphatic endothelial cells (DsRed-iLEC; lower panels), for 24 h. (**g** and **h**) Bar graphs showing quantitative assessment of the capacity of EpXT cells to migrate toward beads coated with iLEC versus MS1 cells (**g**) and SV-LEC versus MS1 cells (**h**). *P<0.05, ***P<0.001.

Next we wanted to determine whether the enhanced capacity of EMT cells to disseminate through the lymphatic system was a result of their overall migratory properties or, whether this reflected a more specific capacity to migrate through the lymphatic system. We used confocal microscopy to examine the presence and localization of blood and lymphatic vessels in EpXT footpad tumors. Blood vessels (CD31+/LYVE-1-) were present in all parts of EpXT footpad tumors (Figure 2a). In contrast, lymphatic vessels (CD31+/LYVE-1+) were clustered in certain regions of the tumors. Further analysis indicated that GFP-EpXT cells displayed a more invasive morphology in areas where lymphatic vessels were present (Figures 2b and c), and demonstrated how tumor cells intravasated into lymphatic vessels in such invasive regions (Supplementary Figure 2). Quantification verified that lymphatic vessel density was significantly higher in invasive compared with noninvasive areas (Figure 2d). In contrast, blood vessel density was decreased in invasive compared with noninvasive areas (Figure 2e).

These data indicated that although EpXT cells appear elongated and migratory in culture, their behavior *in vivo* was different in different regions of the tumor and linked to the presence of lymphatic vessels. To study this further, we developed a 3D co-culture system, which is based on a previously established fibrin beads assay.^23^ GFP-labeled EpXT, EpRas or EpH4 cells were coated onto polymeric beads that were mixed at a 1:1 ratio in a fibrin matrix together with beads coated with dsRed-labeled immortalized human lymphatic endothelial cells (iLEC), or mouse vascular endothelial cells (MS1). Time-lapse movies taken over 24 h demonstrated how individual spindle-like GFP-EpXT cells detached from beads and migrated into the matrix (Supplementary Movie 1). After the initial phase of detachment, GFP-EpXT cells were organized into invasive protrusions and migrated in a more collective fashion toward beads coated with DsRed-iLEC cells. In contrast, EpH4 cells did not migrate at all in this assay (Supplementary Movie 2). Quantification of immunofluorescence images taken at 24 h after seeding the beads showed that 30% of DsRed-iLEC-coated beads were covered with GFP-EpXT cells (Figures 2f and g). When the assay was repeated with beads coated with GFP-EpXT and DsRed-MS1 cells, only 8% of MS1 beads were covered with GFP-EpXT cells. Thus, GFP-EpXT cells migrated 3.75-fold more efficient toward beads coated with DsRed-iLEC cells compared with DsRed-MS1 cells. GFP-EpXT cells also migrated significantly more efficient toward mouse lymphatic endothelial cells (SV-LEC) compared with MS1 cells (Figure 2h). GFP-EpRas cells migrated 1.8-fold more efficiently toward beads coated with DsRed-iLEC cells and 1.2-fold more efficiently toward beads coated with DsRed-MS1 cells compared with nontreated GFP-EpRas cells after treatment with TGF-β1 (Supplementary Figures 3a and b). Together with the *in vivo* results, these data indicated that mammary tumor cells that undergo TGF-β1-induced EMT gain the capacity to migrate in a targeted manner toward lymphatic endothelial cells.

### TGF-β1-induced EMT promotes tumor cell migration toward lymphatic endothelial cells via activation of CCR7

**Figure 3:**
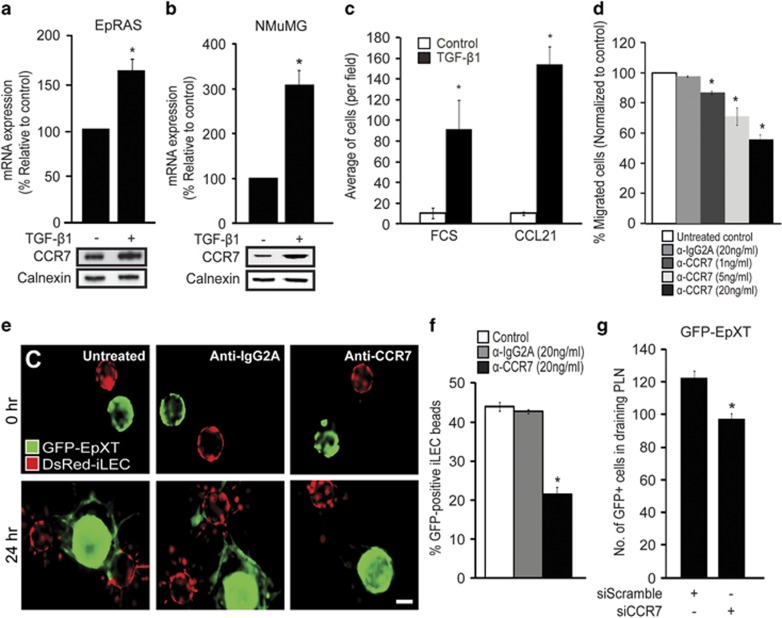
TGF-β1-induced EMT promotes tumor cell migration toward lymphatic endothelial cells via CCR7/CCL21-mediated chemotaxis. (**a** and **b**) Results from expression analysis of CCR7 mRNA and protein levels in TGF-β1 treated (10 ng/ml, 48 h) versus nontreated EpRas cells (**a**), and NMuMG cells (**b**) as determined by qPCR (bar graphs) and immunoblotting (lower panels). (**c**) Bar graph showing quantitative assessment of the capacity of NMuMG cells to invade through matrigel and migrate toward a gradient of fetal calf serum (FCS) or CCL21 in invasion assays. (**d**) Bar graph showing dose-dependent inhibition of migration of NMuMG cells toward CCL21 in the presence of a neutralizing antibody to CCR7. An isotype-matched antibody (IgG2A) was used as a control. (**e**) Representative confocal immunofluorescence images showing the effect of a neutralizing antibody to CCR7 (20 ng/ml) on the capacity of GFP-EpXT cells to migrate toward beads coated with iLEC. (**f**) Quantitative results showing the effect of a neutralizing CCR7 antibody (20 ng/ml) on the capacity of EpXT cells to migrate toward lymphatic endothelial cells (iLEC) in the fibrin beads assay. (**g**) Bar graph showing quantitative assessment of the capacity of GFP-EpXT cells transfected with siRNA against CCR7 (siCCR7), or a scrambled siRNA (siScramble), to disseminate to PLN at 2 days after injection of cells into the footpad of BALB/c mice. Scale bar, 100 μm. Calnexin was used as a loading control in immunoblotting experiments. *P<0.05.

Next we investigated the possible mechanisms that could be involved in directing tumor EMT cells toward lymphatic vessels. On the basis of the documented expression of CCR7 in breast cancer cells and its established role in mediating homing of DCs to lymph nodes, we speculated that CCR7 could have a role in guiding EMT cells toward lymphatic vessels. In line with this, we found that CCR7 was induced during TGF-β1-induced EMT in EpRas cells (Figure 3a), and expressed at higher levels in EpXT versus EpH4 cells (Supplementary Figure 3c). To study this further, we also included an established, frequently used model of transient TGF-β1-induced EMT in Namru mammary gland (NMuMG) epithelial cells. Induction of EMT by treatment with TGF-β1 resulted in significant induction of CCR7 expression also in NMuMG cells (Figure 3b).

This suggested that tumor cells that undergo TGF-β1-induced EMT gain the capacity to migrate toward CCR7 ligands, such as CCL21, which is known to be produced by lymphatic endothelial cells and to promote migration of DCs toward lymphatic vessels.^24^ To test this, we performed invasion assays, in which cells with different EMT properties were seeded in the upper wells and recombinant CCL21 (350 ng/ml), or 10% fetal calf serum, was added in the lower chamber as a chemoattractant. NMuMG cells that were induced to undergo EMT with TGF-β1 migrated significantly more efficient than nontreated cells against fetal calf serum, and even more efficient against CCL21 (Figure 3c). Similar to this, TGF-β1-treated EpRas cells migrated more efficiently toward CCL21 compared with nontreated cells (Supplementary Figure 3d). To investigate the relative contribution of CCR7 to the migration of EMT cells toward CCL21, we performed blocking experiments using an anti-CCR7-neutralizing antibody. In the presence of this antibody, but not an isotype-matched control antibody (IgG2A), the capacity of TGF-β1-treated NMuMG cells to migrate toward CCL21 was inhibited in a dose-dependent fashion (Figure 3d).

Further analysis showed that EpXT cells migrated less efficiently toward DsRed-iLEC in beads assays in the presence of the anti-CCR7-neutralizing antibody (Figures 3e and f). To evaluate the role of CCR7 in mediating lymphatic dissemination of EMT cells *in vivo*, we introduced CCR7 small interfering RNA (siRNA) into GFP-EpXT cells and injected cells into the mouse footpad. GFP-EpXT-siCCR7 cells expressed decreased levels of CCR7 (Supplementary Figure 3e), and migrated less efficient to draining lymph nodes compared with GFP-EpXT-sicontrol cells (Figure 3g).

### CCR7 is regulated by p38 signaling via JunB during TGF-β1-induced EMT

**Figure 4:**
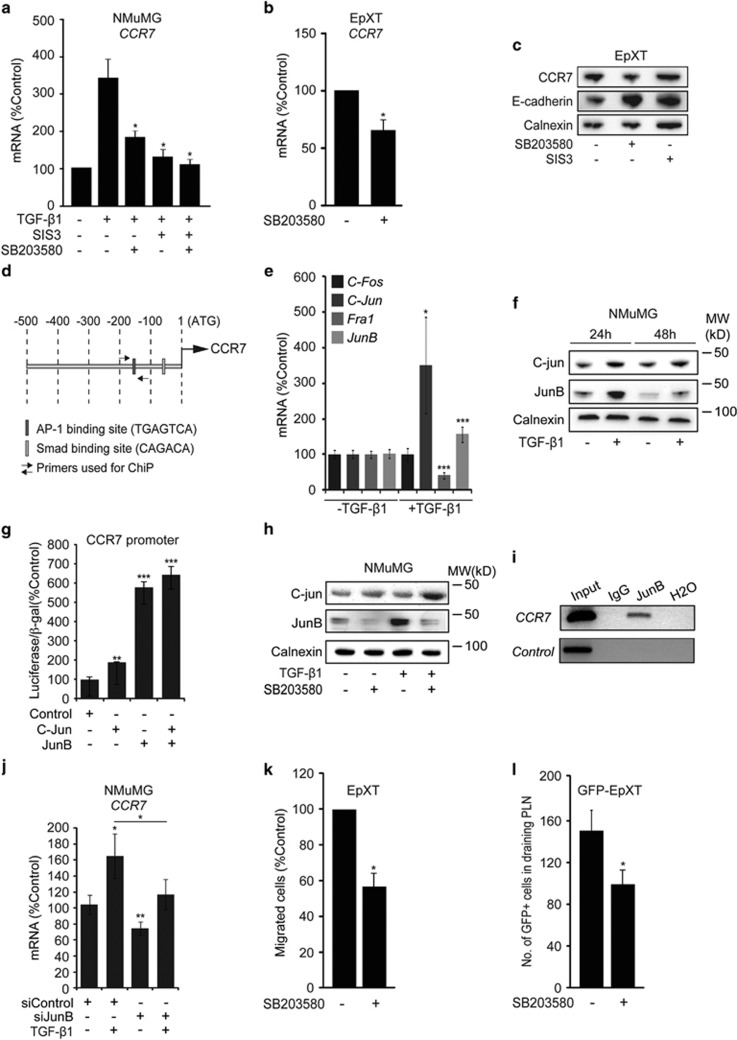
Role of p38 MAPK signaling and the AP-1 factor JunB in the regulation of CCR7 during TGF-β1-induced EMT. (**a**) Bar graph showing the effects of inhibitors of Smad3 (SIS3, 15 μM) and p38 MAPK (SB203580, 20 μM) on the induction of CCR7 after 48 h of TGF-β1-induced EMT (2 ng/ml of TGF-β1) in NMuMG cells. (**b**) Bar graph showing the effect of SB203580 (20 μM, 48 h) on the expression of CCR7 mRNA relative to control (cells treated with vehicle). (**c**) Immunoblot showing the effect of SB203580 (20 μM, 48 h) and SIS3 (15 μM, 48 h) on the expression of CCR7 and E-cadherin in EpXT cells. (**d**) Schematic drawing of the CCR7 promoter showing the location of AP-1 sites and binding sites for primers used for chromatin immunoprecipitation assays. (**e**) Bar graph showing changes in mRNA expression of the AP-1 factors *c-Fos*, *c-Jun*, *Fra1* and *JunB* in NMuMG cells during TGF-β1-induced EMT. (**f**) Immunoblot analysis of the effect of TGF-β1 on the expression of c-jun and JunB in NMuMG cells. (**g**) Results from reporter assays showing the effect of overexpression of C-jun and JunB on the activity of the CCR7 promoter. (**h**) Immunoblotting results showing the effect of TGF-β1 (2 ng/ml) on the expression of C-jun and JunB in NMuMG cells in the absence or presence of SB203580 (20 μM) after 48 h. (**i**) RT–PCR results from chromatin immunoprecipitation assays showing binding of JunB to the CCR7 promoter in TGF-β1-treated NMuMG cells. No binding of JunB was detected in a region lacking consensus AP-1-binding sites (control). (**j**) Bar graph showing the effect of overexpression of siRNA against JunB (siJunB) or control (siControl) on the induction of CCR7 mRNA expression in NMuMG cells during TGF-β1-induced EMT. (**k** and **l**) Bar graphs showing the effect of pretreatment with SB203580 (20 μM, 48 h) on the migration of GFP-EpXT cells toward CCL21 in invasion assays (**k**), and the dissemination of GFP-EpXT cells to draining PLN at 2 days after injection into the footpad of BALB/c mice (**l**). Calnexin was used as a loading control in immunoblotting experiments. *P<0.05, **P<0.01, ***P<0.001.

Next we wanted to determine the mechanism by which CCR7 is regulated during TGF-β1-induced EMT. First, we used a specific inhibitor of Smad3 (SIS3) and a selective inhibitor (SB203580) of p38 MAPK to elucidate the role of Smad-dependent versus P38 MAPK-dependent pathways. We found that the induction of CCR7 mRNA was inhibited in the presence of both Smad3 and p38 MAPK inhibitors during TGF-β1-induced EMT in NMuMG cells (Figure 4a), as well as in EpRas cells (Supplementary Figure 4a). Inhibition of p38 MAPK for 24 h resulted in the downregulation of CCR7 expression in EpXT cells (Figures 4b and c). On the contrary, Smad3 inhibition in EpXT cells had no effect on CCR7 protein levels (Figure 4c), and actually resulted in increased CCR7 mRNA levels (Supplementary Figure 4b). These results prompted us to perform promoter assays to determine whether Smads could activate the CCR7 promoter. Overexpression of Smad3 and Smad4 was not sufficient to activate the CCR7 promoter in NMuMG cells (Supplementary Figure 4c).

These data indicated that Smads were not direct activators of CCR7 and that factors operating downstream of p38 MAPK signaling appeared to be involved in inducing CCR7 expression in EMT cells. Related to this, previous data have shown that CCR7 mRNA expression in monocytes is controlled by p38 MAPK signaling.^25^ The CCR7 promoter contains binding sites for AP-1 factors (Figure 4d), and such factors have been shown to promote CCR7 expression in Hodgkins lymphoma and metastatic squamous carcinoma cells of the head and neck.^26, 27^ On the basis of this, and the fact that AP-1 factors are activated during, and important for the induction of TGF-β1-induced EMT,^17, 28^ we hypothesized that specific AP-1 factors could be involved in inducing CCR7 expression during TGF-β1-induced EMT. In particular, we were interested to study the role of c-Fos, c-Jun, Fra1 and JunB, AP-1 factors that have been implicated in promoting the invasive capabilities of breast cancer cells in response to TGF-β1.^29^ Gene expression analysis showed that among these AP-1 factors, c-Jun and JunB were significantly induced at the mRNA level after 24 h of TGF-β1-induced EMT in NMuMG cells (Figure 4e). In contrast, c-Fos expression did not change, whereas Fra1 was downregulated. Immunoblotting analysis confirmed the induction of c-Jun and JunB in NMuMG cells after both 24 and 48 h of TGF-β1 exposure (Figure 4f). We then used reporter assays to examine the capacity of c-Jun and JunB to activate the CCR7 promoter in NMuMG cells. Overexpression of JunB resulted in potent (sixfold) activation of the CCR7 promoter, whereas the overexpression of c-Jun was less efficient (twofold; Figure 4g).

Next we studied whether the induction of c-Jun and JunB during TGF-β1-induced EMT was mediated via the P38 MAPK pathway. We found that in the presence of the P38 MAPK inhibitor the induction of JunB, but not c-Jun, was inhibited in NMuMG cells (Figure 4h). Similar to this, P38 MAPK signaling was important for *JunB* induction during TGF-β1-induced EMT in EpRas cells, and for JunB expression in EpXT cells (Supplementary Figures 4d and e). The results pointed to a role for p38 MAPK-mediated activation of JunB as a mechanism of CCR7 induction during TGF-β1-induced EMT. In support of this, further studies using chromatin immunoprecipitation assays confirmed binding of JunB to a region of the CCR7 promoter containing an AP-1 site (Figure 4i), and showed that siRNA-mediated knockdown of JunB inhibited the induction of CCR7 during TGF-β1-induced EMT in NMuMG cells (Figure 4j).

The results suggested that the inhibition of P38 MAPK signaling in EMT cells might inhibit their capacity to disseminate through the lymphatic system. To test this, we pretreated GFP-EpXT cells with SB203580 for 48 h and then evaluated the effect on their migratory behavior. GFP-EpXT cells that had been pretreated with the P38 MAPK inhibitor migrated significantly less efficient toward CCL21 in invasion assays (Figure 4k), and had a decreased capacity to disseminate to lymph nodes (Figure 4l) compared with nontreated cells.

### CCL21 expression in lymphatic endothelial cells is induced by TGF-β1 and promotes chemotaxis of EMT cells in a paracrine fashion

**Figure 5:**
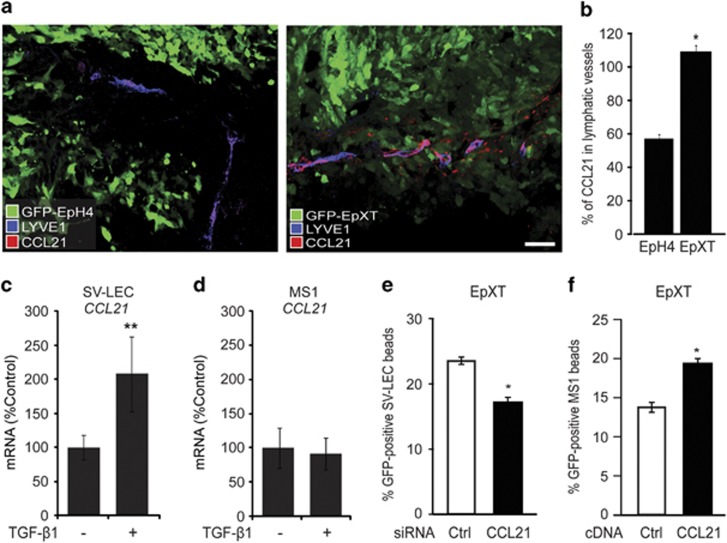
CCL21 expression in lymphatic endothelial cells is regulated by TGF-β1 and important for chemoattraction of tumor EMT cells. (**a**) Representative confocal immunofluorescence images showing staining of CCL21 in LYVE-1-positive lymphatic vessels in GFP-EpH4 and GFP-EpXT footpad tumors at day 6. (**b**) Bar graph showing quantitative assessment of the expression of CCL21 in endothelial cells of lymphatic vessels in GFP-EpH4 versus GFP-EpXT footpad tumors at day 6 after footpad injections. (**c** and **d**) Bar graphs showing the effect of TGF-β1 treatment (10 ng/ml, 24 h) on the expression of CCL21 in SV-LEC cells (**c**) and in MS1 cells (**d**). (**e**) Bar graph showing the effect of overexpression of CCL21 or control siRNA in SV-LEC cells on their capacity to support chemotactic migration of EpXT cells in beads assays. (**f**) Bar graph showing the effect of overexpression of CCL21 or control cDNA in MS1 cells on their capacity to support chemotactic migration of EpXT cells in beads assays. *P<0.05, **P<0.01.

We reasoned that an additional mechanism by which TGF-β1 could promote CCR7/CCL21-mediated chemotaxis of EMT cells toward lymphatic vessels would be to regulate CCL21 expression in lymphatic endothelial cells. In support of this, we found that CCL21 was expressed at increased levels in lymphatic vessels in tumors formed by GFP-EpXT cells, which express endogenous TGF-β1, compared with tumors formed by GFP-EpH4 cells (Figures 5a and b). Further studies showed that CCL21 mRNA levels were significantly induced in both mouse (SV-LEC) and human (iLEC) lymphatic endothelial cells, but not in vascular (MS1) endothelial cells upon TGF-β1 exposure (Figures 5c and d and Supplementary Figure 3f).

These data showed that CCL21 production by lymphatic endothelial cells could be upregulated by TGF-β1 and possibly promote chemoattraction of CCR7-expressing tumor EMT cells. To study this, we knocked down CCL21 in SV-LEC cells and then performed the beads assay with EPXT cells. Indeed, knockdown of CCL21 resulted in less efficient migration of EpXT cells toward SV-LEC beads (Figure 5e). On the contrary, overexpression of CCL21 in MS1 cells enhanced the capacity of these cells to promote targeted migration of EpXT cells (Figure 5f).

### The expression of CCR7 and CCL21 is linked to EMT in human breast cancer

**Figure 6:**
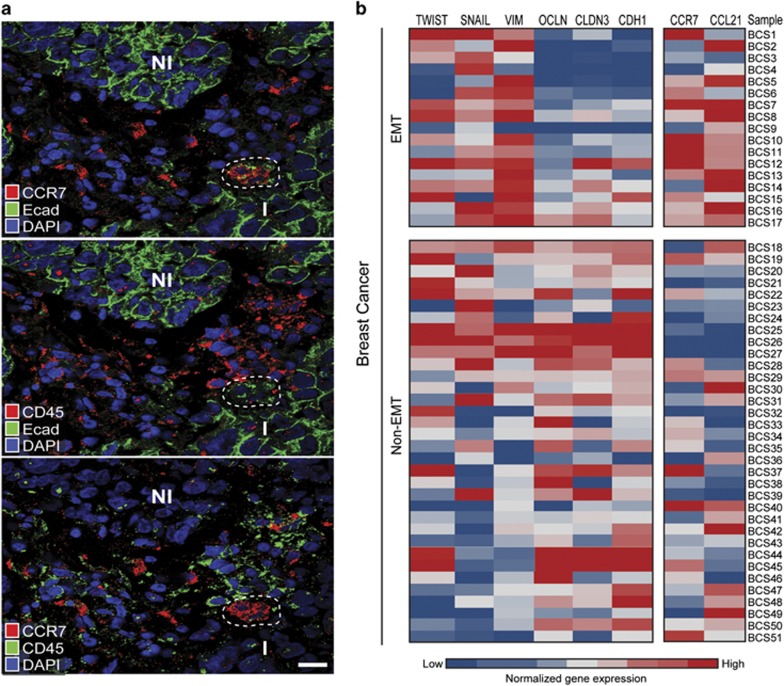
The expression of CCR7 and CCL21 is linked to EMT in human breast cancer. (**a**) Representative confocal immunofluorescence images showing the presence of E-cad^low^/CCR7^pos^/CD45^neg^ tumor cells (outlined area) within an invasive area (I) of a sample of human breast cancer. Tumor cells in noninvasive areas (NI) were more strongly positive for E-cadherin and were negative for CCR7. (**b**) Profiling of 51 human breast cancers based on gene expression data from the Gene Expression Atlas at the European Bioinformatics Institute (EMBL-EBI). Three mesenchymal genes (*TWIST*, *SNAIL* and *VIM*) and three epithelial genes (*OCLDN*, *CLDN3* and *CDH1*), which are induced and repressed, respectively, during EMT were selected to identify tumors with an EMT genotype. The expression of *CCR7* and *CCL21* was higher in the EMT samples compared with non-EMT samples (*P*<0.05 for both).

Finally, we investigated whether the expression of CCR7 and CCL21 could be linked to EMT in human breast cancer. We performed triple staining for E-cadherin, CCR7 and CD45, a pan-immune cell marker, which was used to distinguish CCR7-positive EMT cells from tumor-associated immune cells. Confocal microscopy analysis revealed the presence of invasive (I) areas where tumor cells expressed detectable, but lower levels of E-cadherin compared with tumor cells in noninvasive (NI) areas (Figure 6a). Positive staining for CCR7 was detected in tumor cells within such invasive areas but not in tumor cells in noninvasive areas. The CCR7-positive tumor cells were negative for CD45. In contrast, cells in the adjacent tumor stroma stained positive for CD45.

We also used published microarray data and performed meta-analysis of 51 samples of human breast cancer. We determined the ratio of the values for three mesenchymal genes (TWIST, SNAIL and VIM) and three epithelial genes (OCLDN, CLDN3 and CDH1) known to be induced or, repressed during EMT, respectively. Tumor samples with a mesenchymal/epithelial ratio of more than 1.0 were defined as tumors displaying an EMT profile. Out of 51 tumors, 17 tumors displayed such an EMT profile (Figure 6b). The expression of CCR7 and CCL21 was significantly higher in tumors with an EMT profile compared with tumors with a non-EMT profile.

## Discussion

**Figure 7:**
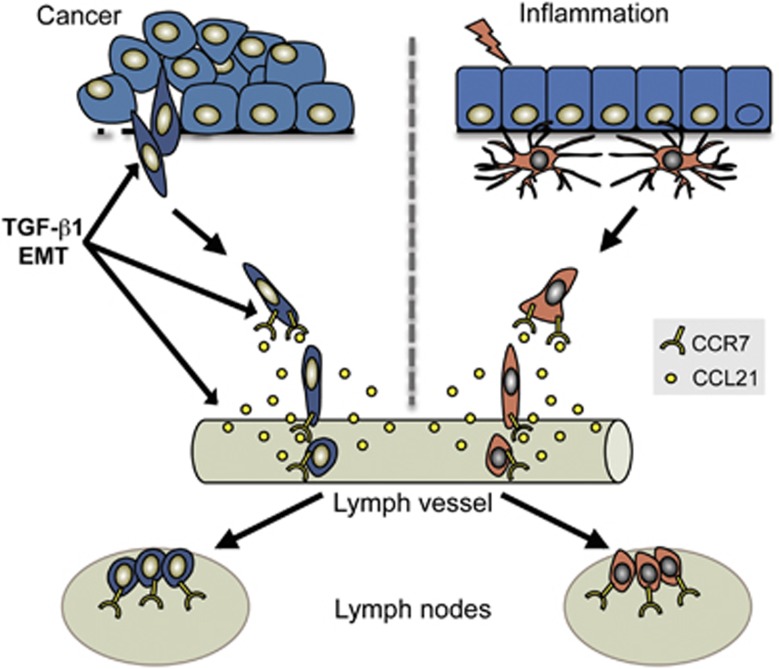
Schematic diagram summarizing the results that indicate that tumor cells undergoing TGF-β1-induced EMT become activated for targeted migration through the lymphatic system, similar to DCs during inflammation. Induction of CCR7 provides both EMT cells and DCs with a capacity to sense and migrate toward a gradient of CCL21, which is produced by lymphatic endothelial cells. TGF-β also induces the expression of CCL21 in lymphatic endothelial cells, which may further promote CCR7/CCL21-mediated migration of EMT cells toward lymphatic vessels.

The findings presented in this study describe TGF-β-induced EMT as a mechanism, which activates tumor cells for targeted dissemination through the lymphatic system. This resembles how DCs become activated and migrate via peripheral lymphatic vessels to lymph nodes during inflammation (Figure 7).^30^ The capacity of both EMT cells and DCs to migrate to lymph nodes is dependent on the induction of CCR7, which provides cells with the capacity to sense and migrate toward CCL21, which is released from lymphatic endothelial cells. Thus, by undergoing EMT tumor cells may hijack a migratory system, which normally is used by DCs to specifically gain access to the lymphatic system. This provides novel insight into a major question in cancer biology, namely why tumor cells frequently disseminate through the lymphatic system.

During recent years, EMT has been increasingly accepted as a developmental process, which is reactivated in breast cancer and other types of cancer, and provides tumor cells with invasive, migratory and cancer stem cell properties.^7, 8, 9, 31, 32, 33^ However, it has not been clear how EMT cells make use of their migratory properties, *in vivo*. Conceptually, our results indicate that tumor cells undergoing EMT may not only become more migratory, in general, but may also acquire the capability to sense and migrate in a targeted fashion toward chemokines released by lymphatic vessels. The implication of this is that EMT may not be the sole factor that determines whether tumor cells actually will migrate away from the primary tumor. Instead, EMT may be regarded as an activation step, which prepares cells for migration. Whether tumor cells actually will migrate, or not, and how they will migrate, will depend on which chemotactic signals that are present in the tumor microenvironment and that can be sensed by the EMT cells. This notion was supported by our findings showing that although EpXT cells are elongated when grown in culture under standard conditions, they only displayed a migratory morphology in certain areas, *in vivo*. Such areas were found to contain a much denser population of lymphatic vessels compared with the rest of the tumors. In contrast, blood vessels were more equally abundant in all parts of tumors. Together with the data from the 3D migration assays, this suggests that mammary tumor cells undergoing TGF-β-induced EMT gain the capacity to migrate toward lymphatic vessels compared with blood vessels. In line with this, recent data show that lymph vessel invasion, but not blood vessel invasion, is a prognostic factor in breast cancer.^3, 34^

Our results showed that CCR7 is regulated during TGF-β-induced EMT and that blockade or knockdown of CCR7 inhibits the capacity of EMT cells to migrate toward CCL21 and lymphatic endothelial cells, and to disseminate through the lymphatic system. These data are in line with other reports showing that the expression of CCR7 is associated with poor prognosis and lymph node metastasis in breast cancer.^35, 36, 37^ Recently, Shields *et al.*^38^ reported on a role for autologous, CCR7-mediated chemotaxis of tumor cells toward lymphatic endothelial cells in 3D matrices. Recently, the expression of CXCR5, another chemokine receptor involved in homing of DCs to lymph nodes, and its ligand CXCL13, was linked to EMT and lymph node metastasis in breast cancer.^39^ Similar to this, others have found that migratory tumor cells may co-opt functional and migratory properties of macrophages in mammary tumors.^40, 41^

Induction of CCR7 was dependent on both Smad and p38 MAPK pathways. Yet, the inhibition of p38 MAPK signaling was more efficient than Smad3 inhibition to decrease CCR7 expression in stable EMT cells. It was also sufficient to inhibit the capacity of EMT cells to disseminate through the lymphatic system. Although p38 MAPK signaling is frequently overactivated in breast cancer,^42, 43^ and also, known as one of the most important non-Smad pathways in EMT,^10, 12, 17, 44^ its role in lymphatic dissemination of tumor cells has not been investigated previously. Intriguingly, P38 MAPK signaling is important for DC migration and homing to LN.^45^ A possible explanation to why p38 MAPK signaling may stand out as a major pathway regulating migratory properties of EMT cells is that it receives input from several pathways that are known to cooperate to induce EMT including TGF-β, Ras and Wnt.^10, 12, 17, 44^ Thus, although Smad signaling primarily is regulated downstream of TGF-β, p38 MAPK signaling serves as a hub, which is co-activated and connects input signals from several EMT-inducing pathways. These results hold the promise of targeting p38 MAPK signaling to inhibit lymph metastasis in breast cancer.

In summary, our results identify TGF-β-induced EMT as a process, which activates tumor cells for targeted migration through the lymphatic system through the induction of DC properties. Considering the fact that activated immune cells are the only cells in our bodies that have a similar capacity as metastatic cancer cells to migrate in and out of vessels and reach distant sites it will be interesting to further penetrate immune cell-like features of tumor cells undergoing EMT and how they are used during metastasis.

## Materials and methods

### Cell culture

Namru Murine Mammary gland (NMuMG), EpH4, EpRAS and EpXT cells were cultivated as described previously.^22, 46^ The murine pancreatic islet endothelial cell line, MS1 and the human immortalized lymphatic endothelial cells (iLEC) were maintained in Endothelial Cell Basal Medium MV 2 containing ascorbic acid, human recombinant basic fibroblast growth factor, human recombinant epidermal growth factor, vascular endothelial growth factor, Long R3 IFG-1, hydrocortisone, 5% fetal calf serum (PromoCell, Germany) and 1% penicillin–streptomycin. The mouse lymphatic endothelial cell line SV-LEC was obtained from Dr Jonathan S. Alexander (Louisiana State University Health Sciences Center, Shreveport, Louisiana) and cultured in DMEM with high glucose supplemented with 10% FBS and 1% penicillin–streptomycin. All cells were cultured in 37 °C and 5% humidified CO_2_ incubator.

For co-culture experiments, GFP-labeled EpH4, EpRAS and EpXT cells were generated by lentiviral transduction of GFP control lentiviral particles (Santa Cruz Biotechnology Inc). DsRed-labeled MS1, iLECand SV-LEC were produced by transduction with mCherry encoding lentiviral particles (Capital Biosciences Inc.).

To investigate the effect of TGF-β1 on EpH4 and EpRAS cells, EpH4 and EpRAS cells were treated with 10 ng/ml of TGF-β1 (R&D Systems, Abingdon, UK). TGF-β1 long-term-treated EpRAS cells were maintained cell culture medium containing 10 ng/ml of TGF-β1 for 2 weeks. NMuMG cells were exposed to 2 or 10 ng/ml of TGF-β1 to stimulate EMT. For experiments using inhibitors, NMuMG cells were pretreated with 15 μM of Specific Smad3 inhibitor (SIS3; EMD Biosciences, San Diego, CA, USA) or 20 μM SB203580 (EMD Biosciences) or 20 μM SP600125 (EMD Biosciences) for 2 h followed by TGFβ-1 exposure. EpXT cells were treated with 15 μM of SIS3 or 20 μM of SB203580 or 20 μM SP600125 for 48 h.

### Animals

Female BALB/c mice (8 weeks old) were obtained from the breeding unit at the Department of Microbiology and Tumor biology (MTC) at Karolinska Institutet, Sweden. Six animals were used per group. All animal experiments were performed according to the ethical guidelines of the Swedish agriculture Board (Stockholms Norra Djurförsöksetiska Nämnd) and Karolinska Institutet.

### Mouse footpad model

GFP-labeled EpH4,EpXT, EpRAS treated and untreated with TGF-β1, EpXT-shTGFβ-RII cells were grown in culture flasks, trypsinized, washed and resuspended in phosphate-buffered saline (PBS), and injected at a total number of 1 × 10^6^ cells into the right hind footpads of 8-week-old BALB/c mice. After 1, 2 or 6 days, footpad tissues from the injection sites, and both ipsilateral and contralateral PLN, were isolated and fixed with 4% paraformaldehyde in PBS for 3 h. Tissues were embedded in OCT compound (Bio-Optica, Milan, Italy) and frozen.

For CCR7 inhibition studies, GFP-labeled EpXT cells were transfected with siRNA against CCR7 and scramble control (Dharmacon/GE Healthcare/VWR International, Stockholm, Sweden) for 24 h. Next day, a number of 1 × 10^6^ of EpXT-siCCR7 or EpXT-sicontrol cells were injected into the right hind footpads of 8-week-old BALB/c mice.

For p38 inhibition studies, GFP-labeled EpXT cells were pretreated with 20 μM SB203580 or vehicle (dimethyl sulfoxide) for 48 h before footpad injections. A total number of 1 × 10^6^ GFP-labeled EpXT cells were resuspended in PBS containing 20 μM SB203580, or vehicle, and injected into the right hind footpad of 8-week-old BALB/c mice.

### Human breast cancer samples

Tissue samples from breast cancer patients were collected during surgery and freshly frozen. A clinical pathologist at Umea University Hospital, Sweden classified the samples according to standard PAD protocols. The Research Ethics Review Board (EPN) of Northern Sweden approved all experiments using human tissues.

### Immunofluorescence staining and confocal imaging

Cells grown on coverslips, or cryosections from footpad tissues or PLN, or human breast cancer tissue samples were fixed in 4% paraformaldehyde in PBS for 15 mins at room temperature. Specimens were blocked in incubation buffer (0.1% bovine serum albumin, 5% normal goat serum and 0.1% sodium azide in PBS) for 1 h and then incubated with primary antibodies diluted in incubation buffer at room temperature for 1 h (coverslips) or overnight (cryosections). The following primary antibodies and dilutions were used: mouse anti-E-cadherin (1:1000; clone 36, BD Biosciences, Stockholm, Sweden); rabbit anti-E-cadherin (1:100; Clone 24E10, Cell Signaling/BioNordika, Stockholm, Sweden) as epithelial marker; rabbit anti-N-cadherin, mouse anti-Twist (1:50; Santa Cruz, Heidelberg, Germany), mouse monoclonal anti-SNAIL1^18^ as mesenchymal markers; rabbit monoclonal anti-phosphoSmad3 (1:100; Epitomics Inc., Cambridge, UK) to detect TGFβ signaling; rabbit anti-CCR7 (1:500; clone Y59, Abcam, Cambridge, UK); hamster anti-CD31 (1:500; clone 2H8, Chemicon/Merck Chemicals and Life Science AB, Solna, Sweden); sheep anti-human CD31 (R&D Systems) to stain endothelial cells; rat-anti-LYVE-1 (1:400, Novus Biolabs, Abingdon, UK); rabbit anti-human LYVE-1 (1:100; ReliaTech, Wolfenbüttel, Germany) and CCL21 (1:100; R&D Systems) as lymphatic endothelial marker. Specimens were washed and incubated with fluorescent-labeled secondary antibodies conjugated to FITC, Cy3 or Cy5 (1:400, Jackson ImmunoResearch, West Grove, PA, USA) and mounted in Vectashield mounting media with DAPI (Vectalabs). Staining was visualized with a standard microscope equipped for fluorescence (Nikon Eclipse 800) or with a confocal microscope (LSM-700; Carl Zeiss Microimaging Inc., Stockholm, Sweden). Confocal stack images were captured through LSM Image Software and used to generate 3D projections to visualize the relationship between tumor cells and vessels in the footpad and lymph node tissues.

### Meta-analysis of human breast cancer arrays

Fifty-one publicly available breast cancer data sets based on the A-AFFY-44-Affymetrix GeneChip Human Genome U133 Plus 2.0 array were collected from the E-TABM-276 database (EMBL-EBI). The ratio between the average values for *TWIST*, *SNAIL* and *VIM* and three epithelial genes (*OCLDN*, *CLDN3*, *CDH1*) known to be induced or, repressed during EMT, respectively. Tumor samples with a mesenchymal/epithelial ratio of more than 1.0 were defined as tumors displaying an EMT profile.

### 3D co-culture fibrin beads assay

A 3D fibrin beads assay generated by Nakatsu *et al.*^23^ was modified into a co-culture assay. GFP-labeled EpH4, EpXT, EpRAS treated and untreated with TGF-β1 cells were coated, separately, onto dextran-coated Cytodex-3 beads (GE Healthcare), mixed at a 1:1 ratio with beads coated with DsRed-labeled MS1 or iLEC and embedded into a fibrin matrix in 24-well plates. Twenty-five beads of EMT/non-EMT cells were mixed with 25 beads of MS1/iLEC. Migration of EMT or non-EMT cells toward MS1 or iLEC was monitored under a fluorescence microscope. At 24 h later, the number of beads of MS1 cells, or iLEC, that were covered with GFP-labeled EMT or non-EMT cells were counted. Images were captured by using an Axio Observer Z1 (Carl Zeiss, GmbH). Data were expressed as the percentage of GFP-positive beads.

To block CCR7, beads coated with GFP-labeled EpXT cells were pretreated with a neutralizing monoclonal anti-mouse CCR7 (20 ng/ml) antibody or an IgG2A isotype-matched control antibody (20 ng/ml) for 30 min before the assay. To inhibit CCL21, SV-LEC were transfected with siRNA against CCL21, or scrambled control (Dharmacon) 24 h before the assay. In experiments where gain of function of CCL21 in MS1 cells was studied, an expression vector encoding murine CCL21 (pcDNA3.1–CCL21) was transfected into the MS1 cells 24 h before coating onto the beads. The pcDNA3.1–CCL21 construct was in a previous study shown to promote chemotactic migration of CCR7-positive immune cells after injection into mice,^47^ and was a kind gift from Dr Seonghyang Sohn at the Department of Microbiology School of Medicine, Suwon, Korea. An empty pcDNA3.1 vector was used as a control in these experiments.

### Time-lapse confocal laser scanning

3D co-culture fibrin bead assay was performed as described above in a 12-well glass bottom plate (MatTek Corporation, Ashland, MA, USA). The plate was transfered to a laser confocal microscope system (Zeiss LSM510, equipped with a motorized stage and cultivation system) maintained at 37 ° and 5% CO_2_ with a humidifier. Z slices were acquired using × 10 objective (five to nine slices per field very 15  min using 1% laser capacity for 48 h). Multiwells with different cells or treatments were imaged during one experiment using Multi time series.

### Immunoblotting

Total protein extracts were prepared by lysing cells in Radio Immuno Precipitation Assay buffer (Pierce Biotechnology/Life Technologies Europe BV, Stockholm, Sweden) supplemented with a protease inhibitor cocktail (Roche, Stockholm, Sweden). Equal amounts of total proteins were separated by standard electrophoresis using precasted 4–12% gradient NuPage gels (Invitrogen, Carlsbad, CA, USA). Proteins were transferred onto nitrocellulose membranes, blocked and incubated with primary antibodies at 4 °C overnight. Antibodies used for immunoblotting were: rabbit anti-CCR7 (1:500, clone Y59, Abcam); mouse anti-vimentin (1:500, Abcam); mouse anti-twist (1:200, Santa Cruz); rabbit anti-N-cadherin (1:500, Abcam); mouse anti-E-cadherin (1:1000; clone 36, BD Biosciences); rabbit anti-occludin (1:500, Invitrogen); rabbit anti-calnexin (1:2000); rabbit anti-claudin-3 (1:1000, Zymed/Life Technologies Europe BV, Stockholm, Sweden); rabbit anti-claudin-5 (1:1000, Zymed) rabbit anti-ZO1 (1:1000, Zymed); rabbit anti-Smad3 (1:500, Cell Signaling), rabbit anti-phosphoSmad3 (1:500, Cell Signaling) and rabbit anti-TGF-b (1:500, 56E4, Cell Signaling) Blots were probed with horseradish peroxidase-labeled anti-rabbit or anti-mouse secondary antibodies (1:5000, Cell Signaling) for 1 h. After washing, blots were incubated with ECL solution (Pierce Scientific) for 5 min and signals were detected by using FluoroChem Q (Alpha Innotech, Kasendorf, Germany).

### Quantitative real-time RT–PCR analysis

Total RNA was extracted using RNeasy mini kit (Qiagen, Valencia, CA, USA) according to the manufacturer's instructions. First-strand cDNA synthesis was performed by using the iScript cDNA synthesis kit (Bio-Rad, Solna, Sweden) using an amount of 1 μg of total RNA. For qPCR (quantitative real-time PCR) analysis, 5 ng of the cDNA mixture was used for PCR amplification by KAPA SYBR fast qPCR kit (Kapa Biosystems/Techtum Lab AB, Umeå, Sweden) with validated QuantiTect primers (Qiagen). The following genes were analyzed: CCR7; CCL21; TGFβ-RII, GAPDH and L19. For a list of primers used see Supplementary Table S1 in the Supplementary Information. The PCR was carried out as follows: 3 min at 95 °C followed by 35 cycles of 3 s at 95 ºC, 20 s at 55 °C and 2 s extension step at 72 °C in RotorGene RG-3000A PCR system.

To investigate the effect of TGF-β1 on iLEC, iLEC was serum starved in endothelial cell basal medium containing 0.5% FBS for 24 h before TGF-β1 (10 ng/ml) exposure. After 48 h, RNA was collected and the expression of CCL21 was analyzed as described above. Gene expression analyses were normalized to the expression of GAPDH. To study the effect of SIS3 or p38 inhibition, EpXT cells were treated with 20 μM SB203580 for 24 h before qPCR analysis.

### Luciferase reporter assay

A CCR7 promoter reporter construct was generated to study the capacity of Smad and AP-1 transcription factors to activate the CCR7 promoter in reporter assays. PCR-based cloning (for primer sequences see Supplementary Information) was used to amplify a 500-bp sequence upstream of the transcriptional start site of the CCR7 gene from genomic DNA prepared from 293 cells according to standard protocols. This sequence is known to contain the core CCR7 promoter including a TATA box, a binding site for AP-1 factors^27^ and a binding site for Smads (CAGACA). The amplified promoter sequence was cloned into a pGL3 luciferase reporter vector (Promega Biotech, Nacka, Sweden) and verified for accuracy. Expression vectors encoding C-Jun (kind gift from Dr Nancy Colburn, National Cancer Institute, MD), JunB (kind gift from Dr Anders Sundqvist, Uppsala University), Smad3 and Smad4,^18^ were transfected in combination with the CCR7 promoter luciferase construct into NMuMG cells using Lipofectamine 2000 (Invitrogen), according to standard procedures. A plasmid encoding β-Galactosidase (β-gal) under the cytomegalovirus promoter was used as an internal control for transfection efficiency. Reporter constructs and expression plasmids were transfected Luciferase and β-GAL activities were analyzed using a luciferase assay kit (Biothema, Stocholm, Sweden) and measured with a Polar Star Omega (BMG LABTECH, Cary, NC, USA) plate reader. Luciferase values were normalized to β-GAL values to account for variations in transfection efficiency.

### Chromatin immunoprecipitation

Chromatin was isolated from 10-cm dish confluent cells. First, the cells were washed twice with PBS, crosslinked in 1% paraformaldehyde in PBS for 10 min and quenched with glycine (125 mM in PBS) for 5 min. Cells were scraped in 10 mM Tris (10 mM NaCl, 3 mM MgCl_2_, 1% NP-40, 1% SDS, 0.5% DOC protease inhibitor, pH 8.1) and sonicated on settings 30 s on, 90 s off for 15 times to obtain 250-bp chromatin fragments with a Bioruptor Standard sonicator (Diagenode, Diagnostics, Seraing, Belgium). Sonicated samples were centrifuged at 16 100 *g* for 5 min at 4 °C to remove cell debris. The supernatant was divided in two aliquots, one that were used further for in immunopreciptitation and the other as input. To input sample, 80 μl PBS and 3.5 μl 5M NaCl was added and incubated at 65 °C for 16 h. Immunoprecipitation was done using rabbit-anti-Junb (Santa Cruz) and rabbit-anti-Smad1/2/3 (Santa Cruz) antibodies coupled to Dynabeads (Dynabeads, Life Technologies, Grand Island, NY, USA). The antibody coupling was done according to manufacturer's protocol. The chromatin was diluted to 950 μl in 16.7 mM Tris (167 mM NaCl, 0.01% SDS, 1.1% TX100, pH 8.1) and 0.5 mg antibody-coupled magnetic beads were added, and incubated overnight at 4 °C. The next day the samples were sequentially washed in steps 5  min on a rotator with washing buffer I (20 mM Tris,150 mM NaCl, 2 mM EDTA, 0.1% SDS, 1% TX100 pH 8.1), washing buffer II (20 mM Tris, 500 mM NaCl, 2 mM EDTA, 0.1% SDS, 1% TX100 pH 8.1), washing buffer III (10 mM Tris, 0.25M LiCl, 1 mM EDTA, 1% NP-40, 1% deoxycholate pH 8.1) and washing buffer IV (10 mM Tris, 1 mM EDTA pH 8.1). The samples were eluted in 1% SDS, 0.1M NaHCO3 and incubated 15 min on a roller. To the eluted chromatin, 4 μl 5M NaCl was added and incubated at 65 °C for 16 h. Both input and immunoprecipitated samples were then treated with Proteinase K for 1 h at 45 °C. The samples were purified using PCR purification kit (Qiagen). To amplify the fragments of interest PCR was run with REDTaq Readymix PCR Reaction Mix (Sigma-Aldrich, Stockholm, Sweden) according to the manufacturer's recommendations. PCR products were separated in 2% agarose. All procedures were done at room temperature, unless stated otherwise. All CHIP experiments were repeated three times.

### Invasion assay

Invasion assays were performed by using 12 mm, 8-μm pore cell culture inserts (Millipore, Billerica, MA, USA). A number of 50 000 NMuMG cells, untreated or pretreated with TGF-β1 (10 ng/ml) for 24 h were trypsinized, resuspended in growth factor-reduced Matrigel (2 mg/ml, BD Biosciences) diluted 1:5 in DMEM GlutaMax supplemented with 1% FBS and seeded into the cell culture inserts. For cells pretreated with TGF-β1, TGF-β1 was added to the cell–matrigel suspension. The cell culture inserts were put in 24-well plates in which 400 μl DMEM GlutaMax supplemented with 1% FBS and recombinant mouse CCL21 (350 ng/ml, R&D Systems) had been added.

To study the functionality of CCR7 in mediating chemotaxis of TGFβ-1-treated NMuMG cells toward CCL21, cells were pretreated with a neutralizing anti-CCR7 monoclonal antibody at different doses (1, 5 and 20 ng/ml; MAB3477, R&D systems) and control antibody IgG2A isotype control (20 ng/ml; R&D systems) for 30 min before seeding.

After 16 h, nonmigrated cells were removed from the inserts with a cotton swab and the insert membranes were fixed in methanol. Insert membranes were removed and mounted with Vectashield containing DAPI (Vector Labs/BIONORDIKA, Stockholm, Sweden). The membranes were viewed with using a Nikon Eclipse 800 microscope. Images were captured using a × 10 objective and the number of invaded cells was counted in six random fields.

For EpH4, EpXT, EpRAS treated and untreated with TGF-β1 and EpXT-shRII cells, a number of 25 000 cells were used. To study the effect of p38 inhibition, EpXT cells were pretreated with 20 μM SB203580 for 48 h. The inhibitor was then also added to the cell culture inserts. For these cells, fixation was performed after 6 h and cells were quantified as above.

### Quantitative analyses of cell migration to lymph nodes

Popliteal (footpad model) and axillary (mammary fat pad model) lymph nodes were isolated and longitudinally sectioned. Two midsections from each lymph node were immunofluorescently stained for LYVE-1 according to above and examined using the Nikon Eclipse 800 microscope and Zeiss LSM-700 confocal microscope. Images were captured using a × 20 objective (Nikon Eclipse 800) and the number of GFP-positive cells was counted in three fields per section.

### Quantitative analyses of blood/lymphatic vessel density in human breast cancer samples and footpad tumors

Sections of human breast cancer tissues and footpad tissues were stained with antibodies against LYVE-1 and CD31. Images from three random areas in the human breast cancer tissues and footpads were captured using a Zeiss LSM-700 confocal microscope using × 20, × 10 or × 4 objectives, respectively. Images were taken using the same contrast and bright-field settings and were imported into ImageJ software (http://rsb.info.nih.gov/ij) for measurement of blood/lymphatic vessel area density. Intratumoral lymphatic vessel density in the footpad tissues was quantified by dividing the area of LYVE-1-positive lymphatic vessels with the total tumor area in each image. For quantification of blood/lymphatic vessel density within the invasive and noninvasive region of footpads, images were imported into ImageJ software, three random invasive and noninvasive regions were marked with region of interest manager based on the morphology of the GFP-positive EpXT tumor cells. Regions with elongated tumor cells were defined as invasive regions whereas regions with less elongated tumor cells were define as noninvasive regions. Measurement of blood/lymphatic vessel area density was carried out in the regions marked by region of interest manager. Blood/lymphatic vessel density in the footpad tissues was quantified by dividing the area of LYVE-1-positive/CD31-low lymphatic vessels or CD31-high/LYVE-1-negative blood vessels with the total tumor area in each image.

### Quantitative analyses of CCL21 expression in lymphatic vessels of footpad tumors

Sections of footpad tissues were stained with antibodies against LYVE-1 and CCL21. Images from three random areas in the footpads were captured using a Zeiss LSM-700 confocal microscope and a × 10 objective and imported into ImageJ for measurement of CCL21 area density in lymphatic vessels. Quantification of the percentage of the CCL21-positive area of each lymphatic vessel was performed.

### Quantitative analyses of TGF-β1 signaling and EMT markers in human breast cancer samples

Sections of human breast cancer samples were co-stained with E-cadherin and p-Smad3 to detect TGF-β1 signaling or an EMT cocktail consisted of Snail/Twist to detect EMT *in vivo*. Three random images in human breast cancer samples were captured by using Zeiss LSM-700 confocal microscope and a × 20 objective. Nuclei with positive p-Smad3 or Snail/Twist staining was counted within the tumor regions.

### Statistical analysis

Data represent means±s.e.m. with at least two to three independent experiments in triplicate. For animal experiments, data represent means±s.e.m. with *n*=6 mice per group. Statistical analyses were determined by using Student's *t*-test. *P*<0.05 was considered as significant difference between the groups.
