# Supplementary figures and images for: TGF-β1-induced EMT promotes targeted migration of breast cancer cells through the lymphatic system by the activation of CCR7/CCL21-mediated chemotaxis
Source: Oncogene. 2015 May 11;35(6):748–60. doi: 10.1038/onc.2015.133 (PMC4753256; doi:10.1038/onc.2015.133)

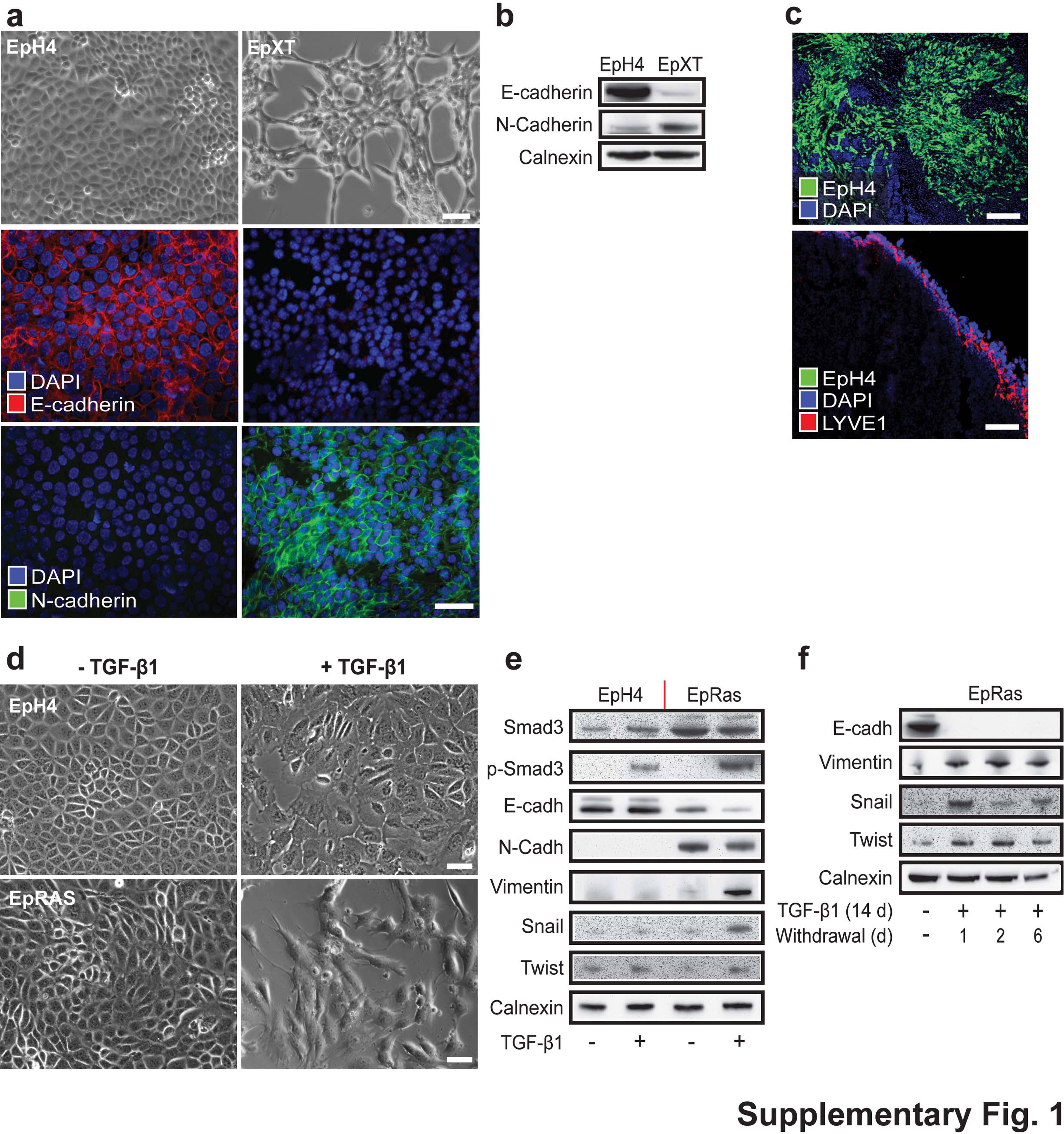

Supplement: Supplementary Figure 1 [file onc2015133x1.tif]

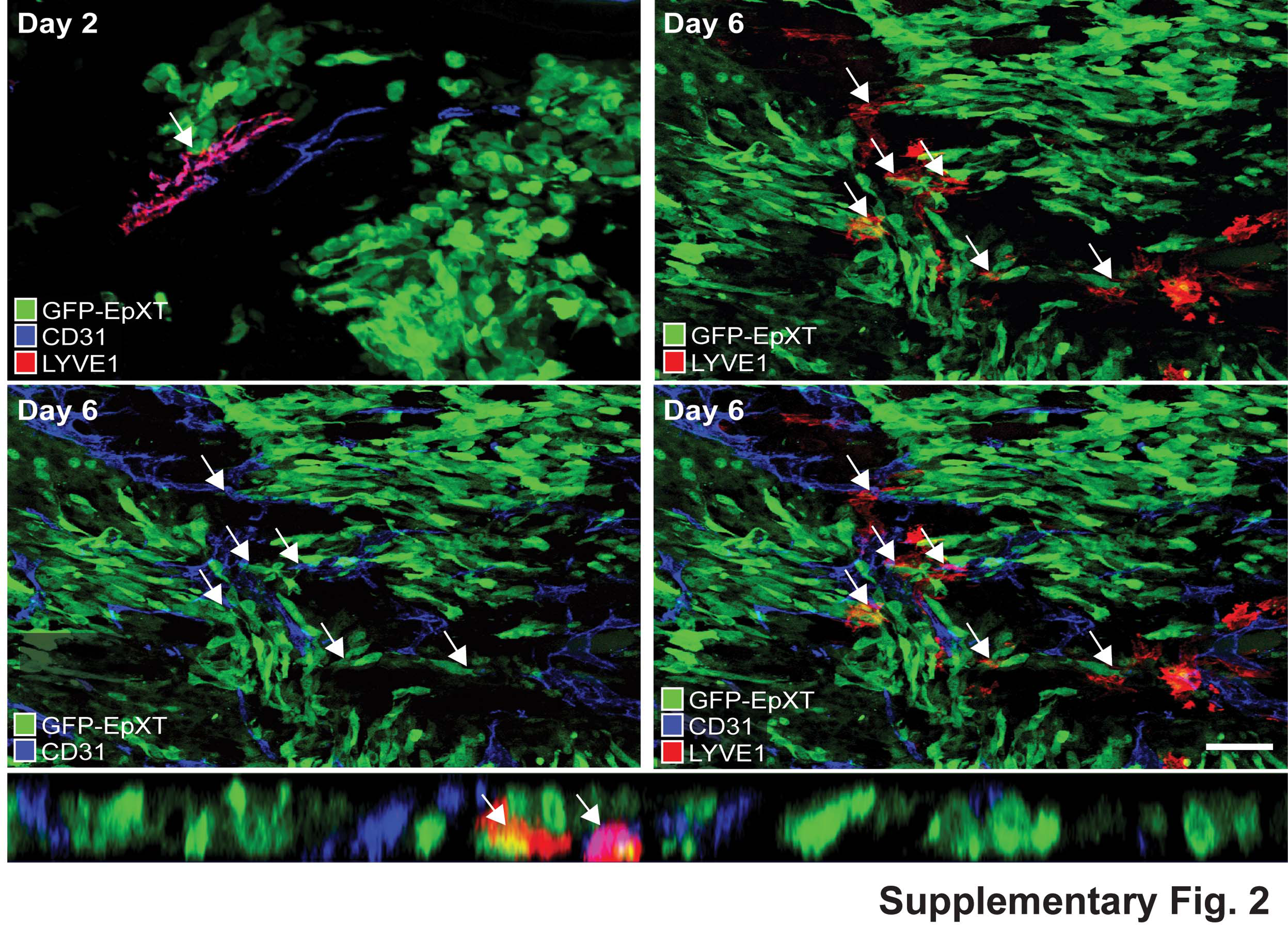

Supplement: Supplementary Figure 2 [file onc2015133x2.tif]

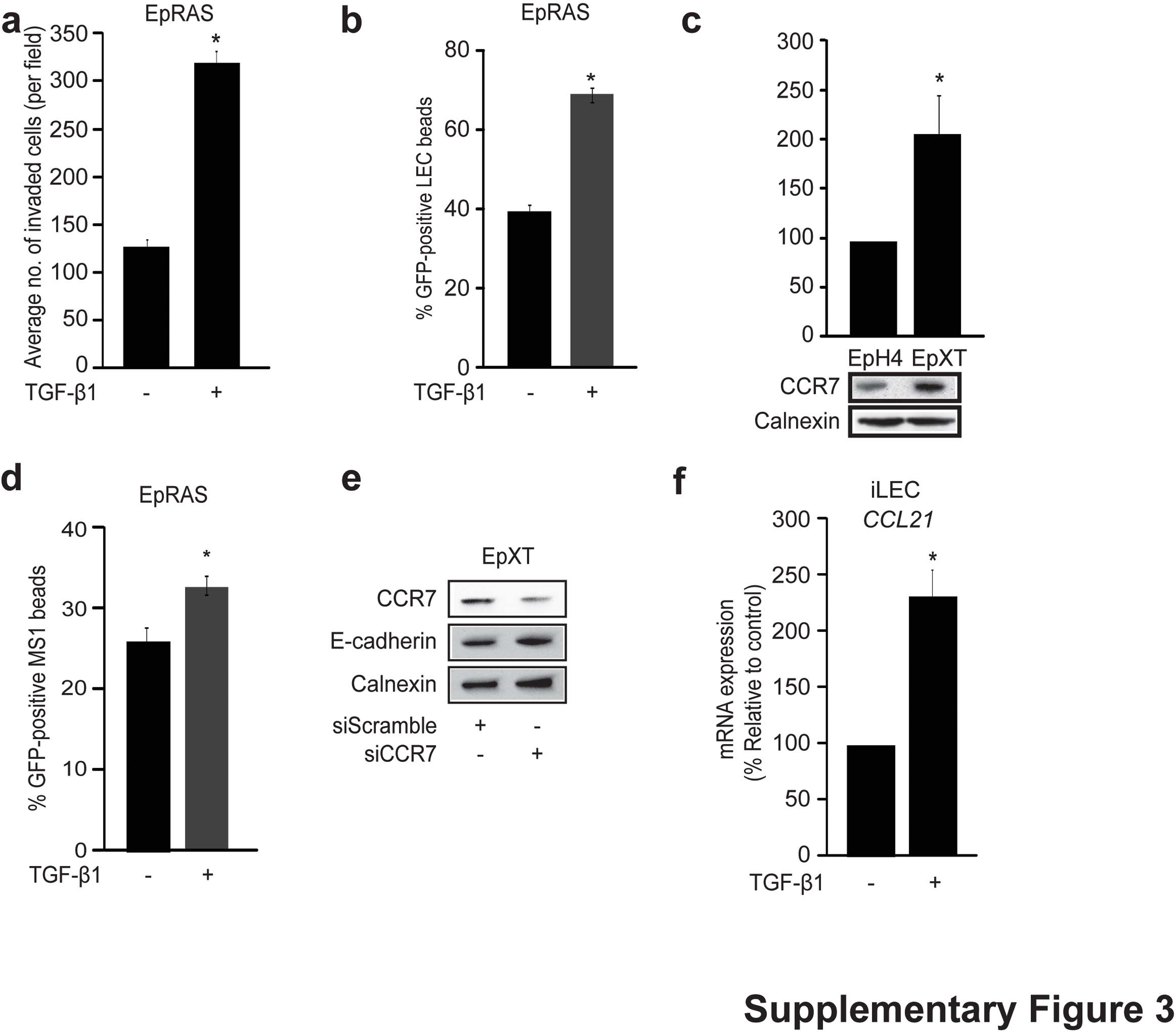

Supplement: Supplementary Figure 3 [file onc2015133x3.tif]

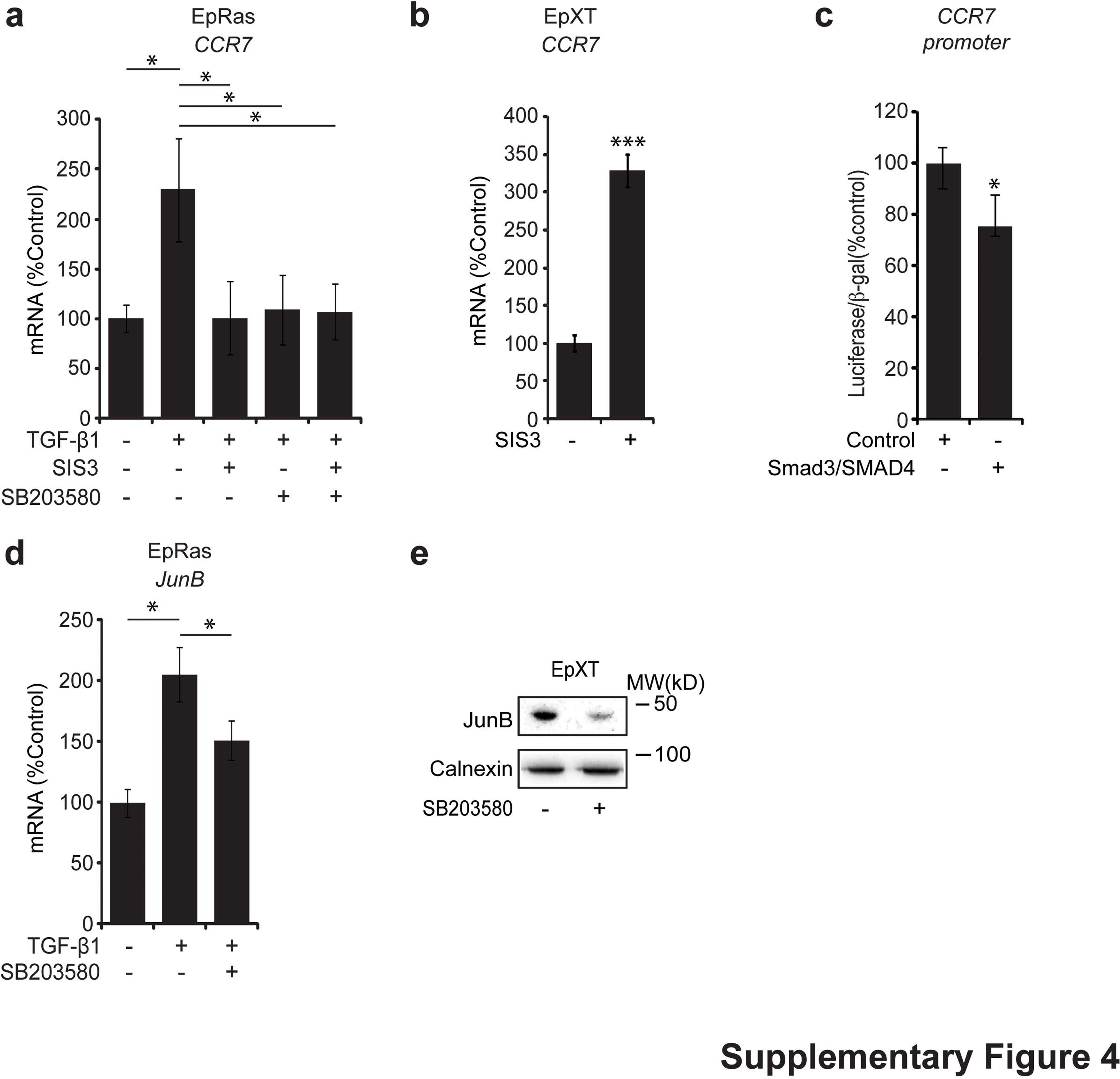

Supplement: Supplementary Figure 4 [file onc2015133x4.tif]
