# Supplementary material for: TGF-β1-induced EMT promotes targeted migration of breast cancer cells through the lymphatic system by the activation of CCR7/CCL21-mediated chemotaxis
Source: Oncogene. 2015 May 11;35(6):748–60. doi: 10.1038/onc.2015.133 (PMC4753256; doi:10.1038/onc.2015.133)
Supplement: Supplementary Information [file onc2015133x5.doc]

### SUPPLEMENTARY INFORMATION

# TGF-β1-induced EMT promotes targeted migration of breast cancer cells through the lymphatic system by activation of CCR7/CCL21-mediated chemotaxis

**Mei-Fong Pang^1, 5^, Anna-Maria Georgoudaki^2^, Laura Lambut^1^, Joel Johansson^1^, Vedrana Tabor^1^, Kazuhiro Hagikura^1, 6^, Yi Jin^1^, Malin Jansson^3^, Jonathan S. Alexander^4^, Celeste M. Nelson^5^, Lars Jakobsson^1^, Christer Betsholtz^1^, Malin Sund^3^, Mikael C. I. Karlsson^2^ and Jonas Fuxe^1, *^**

### Contents:

Page 2: Supplementary Table 1

Page 3-4: Legends to Supplementary Figures 1-3

Page 5: Legends to Supplementary Movies 1-2

**Table S1**: List of primers used for qPCR, RT-PCR and cloning of the CCR7 promoter.

| **Name** | **Catalog number/Sequence** | **Company** |
| --- | --- | --- |
| Human GAPDH | QT01192646 | Qiagen AB, Sollentuna, Sweden |
| Human CCL21 | QT01157366 | Qiagen AB |
| Mouse GAPDH | QT01658692 | Qiagen AB |
| Mouse CCR7 | QT00240975 | Qiagen AB |
| Mouse Ccl21 | QT00314097 | Qiagen AB |
| Mouse C-jun | QT00296541 | Qiagen AB |
| Mouse C-fos | QT00147308 | Qiagen AB |
| Mouse Fra1 | QT00106561 | Qiagen AB |
| Mouse rpl19 | QT00166145 | Qiagen AB |
| Mouse JunB | QT00241892 | Qiagen AB |
| CCR7 promoter cloning fwd | 5’-CTCGAGATCTGAAGGGGGGAG  AAAAAAGATACATCGTG-3’ | Invitrogen |
| CCR7 promoter cloning rev | 5’-TGCCAAGCTTGACGCTCTCTGG  GCGGTAAAACC-3’ | Invitrogen |
| AP-1 CCR7 promoter fwd | 5’-GGCACTACTACCAGGGGCT-3’ | Invitrogen |
| AP-1 CCR7 promoter Rev | 5’-ACCACATTCGGCTGTCATAGG-3’ | Invitrogen |
| Neg. ctrl CCR7 promoter fwd | 5’-CAGAGACAAGAGACCACGCA-3’ | Invitrogen |
| Neg. ctrl CCR7 promoter rev | 5’-CAGGTGTCAGCCTACTTCCA-3’ | Invitrogen |

### Supplementary figure legends

**Supplementary Figure 1.** Different EMT properties of EpXT, EpH4 and EpRas cells.

(**a**) Representative brightfield and immunofluorescence images showing differences in cell morphology, and expression of E-cadherin and N-cadherin in EpH4 versus EpXT cells. Scale bars = 50 μm. (**b**) Immunoblotting analysis of E-cadherin and N-cadherin expression in EpH4 versus EpXT cells. (**c**) Confocal immunofluorescence images showing GFP-EpH4 cells in the mouse footpad (upper image) but not in popliteal lymph nodes (PLN, lower image) at day 6 after injection in syngeneic BALB/c mice. Scale bars = 200 μm (Footpad) and 50 μm (PLN). (**d**) Brightfield images showing the effect of TGF-β1 (10 ng/ml, 48 h) on the morphology of EPH4 and EpRas cells. Scale bars = 50 μm. (**e**) Immunoblotting analysis of the expression of total and phosphorylated Smad3 (pSmad3) levels and various EMT markers in EpH4 cells and EpRas cells at baseline, and after treatment with TGF-β1 (10 ng/ml, 48 h). (**f**) Immunoblotting analysis showing that EpRas cells treated with TGF-β1 (2 ng/ml) for long-term (14 days) maintain an EMT phenotype for at least 6 days after TGF-β1 withdrawal.

Brightfield images showing the effect of TGF-β1 (10 ng/ml, 48 h) on the morphology of EPH4 and EpRas cells. Scale bars = 50 μm.

**Supplementary Figure 2.** TGF-β1-induced EMT promotes targeted migration of tumor cells towards lymphatic vessels. Confocal immunofluorescence images of GFP-EpXT footpad tumours at day 6 after injection. Association of GFP-EpXT cells with lymphatic vessels (CD31-low/LYVE-1-positive) and blood vessels (CD31-high/LYVE-1-negative) is shown. Arrows point to areas of tumour cell migration towards and invasion into lymphatic vessels. The dashed line in the middle panels marks the location of the Z projection in the lower panel. Scale bar = 50 μm.

**Supplementary Figure 3.**

**(a, b)** Bar graphs showing results from quantitative analysis of the effect of TGF-β1 on migration of GFP-EpRas cells towards iLEC (**a**) and MS1 cells (**b**) in beads assays. **(c)** Bar graph showing the expression of CCR7 mRNA and protein levels in EpXT compared to EpH4 cells. (**d**) Bar graph showing the effect of TGF-β1 (10 ng/ml, 48 h) on the capacity of EpRas cells to invade through matrigel and migrate towards a gradient of CCL21 in invasion assays. (**e**) Immunoblotting results showing the effect of siRNA against CCR7 (siCCR7), or a scrambled siRNA (siScramble), on the expression of CCR7 and E-cadherin in EpXT cells. (**f**) Bar graph showing the effect of TGF-β1 treatment (10 ng/ml, 24 h) on the expression of CCL21 in iLEC. Calnexin was used as a loading control in immunoblotting experiments.

**Supplementary Figure 4.**

**(a)** Bar graph showing the effects of inhibitors of Smad3 (SIS3, 15 µM) and p38 MAPK (SB203580, 20 µM), on the induction of CCR7 after 48 h of TGF-β1-induced EMT (2 ng/ml of TGF-β1) in EpRas cells. (**b**) Bar graph showing the effect of the SIS3 inhibitor on the expression of CCR7 mRNA expression in EpXT cells. (**c**) Results from reporter assays showing the effect of overexpression of Smad3 and Smad4 on the activity of the CCR7 promoter in NMuMG cells. (**d**) Bar graph showing the effects of SB203580 (20 µM)

on the induction of JunB after 48 h of TGF-β1-induced EMT (2 ng/ml of TGF-β1) in EpRas cells. (**e**) Immunoblotting results showing the effect of SB203580 (20 µM) on the expression of JunB in EpXT cells. Calnexin was used as a loading control in immunoblotting experiments.

**Supplementary Movie 1.**

Time laps movie taken over 24 h showing beads coated with GFP-EpXT or dsRed-iLEC cells, which were mixed at a 1:1 ratio and embedded in a fibrin matrix. Note how GFP-EpXT cells detach from beads and migrate into the matrix. After the initial phase of detachment, GFP-EpXT cells appear to be organized into invasive protrusions and migrate in a more collective fashion towards beads coated with DsRed-iLEC cells.

**Supplementary Movie 2.**

Time laps movie taken over 24 h showing beads coated with GFP-EpH4 or dsRed-iLEC cells, which were mixed at a 1:1 ratio and embedded in a fibrin matrix. Note how GFP-EpH4 stick to the beads and do not migrate at all in this assay
